# Advances in Extraction Protocols, Degradation Methods, and Bioactivities of Proanthocyanidins

**DOI:** 10.3390/molecules29102179

**Published:** 2024-05-07

**Authors:** Lishan Liang, Yingjie Liu, Liyan Wu, Luo Weng, Honghao Qiu, Wenting Zhong, Fanxin Meng

**Affiliations:** 1 College of Pharmacy and Food Science, Zhuhai College of Science and Technology, Zhuhai 519041, China; lls_773@163.com (L.L.); yjl990911@163.com (Y.L.); wuliyan@zcst.edu.cn (L.W.); wlabwengluo@163.com (L.W.); qiuhh21@mails.jlu.edu.cn (H.Q.); zwt_6249@163.com (W.Z.); 2 College of Life Science, Jilin University, Changchun 130012, China

**Keywords:** proanthocyanidins, flavan-3-ols, extraction, degradation, bioactivities

## Abstract

Proanthocyanidins, natural polyphenolic compounds abundantly present in plants, exhibit diverse bioactivities, including antioxidative, anti-inflammatory, and antibacterial effects. These bioactivities are intricately linked to the degree of polymerization of these compounds. Through a comprehensive analysis of recent domestic and international research, this article synthesizes the latest advancements in the extraction process, degradation methods, as well as the biological activities and underlying mechanisms of proanthocyanidins. Furthermore, future research endeavors should prioritize the refinement of extraction techniques, the elucidation of bioactive mechanisms, and the development of formulations with enhanced potency. This will maximize the utilization of proanthocyanidins across diverse applications.

## 1. Introduction

The natural polyphenolic compounds known as proanthocyanidins (PCs) exist abundantly in various plants, including cinnamon bark (*Cinnamomum zeylanicum*), sorghum grains, grapes (*Vitis vinifera*), apples (*Malus pumila*), and blueberries (*Vaccinium corymbosum*) [1]. Proanthocyanidins are also the main pigment agents of the red, blue, or purple colors in the fruit or flowers of many plants and are primarily found in their skins, flesh, seeds, flowers, and leaves [2,3]. High-purity forms of proanthocyanidins, such as catechin, epicatechin, and epicatechin gallate, often appear as white or near-white powders with a subtle aroma and a slightly astringent taste. These compounds exhibit numerous beneficial properties, including solubility in water, non-cytotoxicity, and low allergenic potential, making them effective antioxidants, anti-inflammatories, and antimicrobials [4]. Their role in defending against both biological and abiotic stressors underscores their significant potential in promoting plant and human health.

In recent years, scholars from both domestic and international circles have conducted extensive research on the extraction processes and biological activities of proanthocyanidins. This article aims to evaluate and compare the various extraction techniques employed in recent years and to summarize and analyze the biological activities of proanthocyanidins and their mechanisms of action in disease prevention and treatment, ultimately aiming to provide insights and directions for further in-depth research.

## 2. The Chemical Structure of Proanthocyanidins

**Figure 1:**
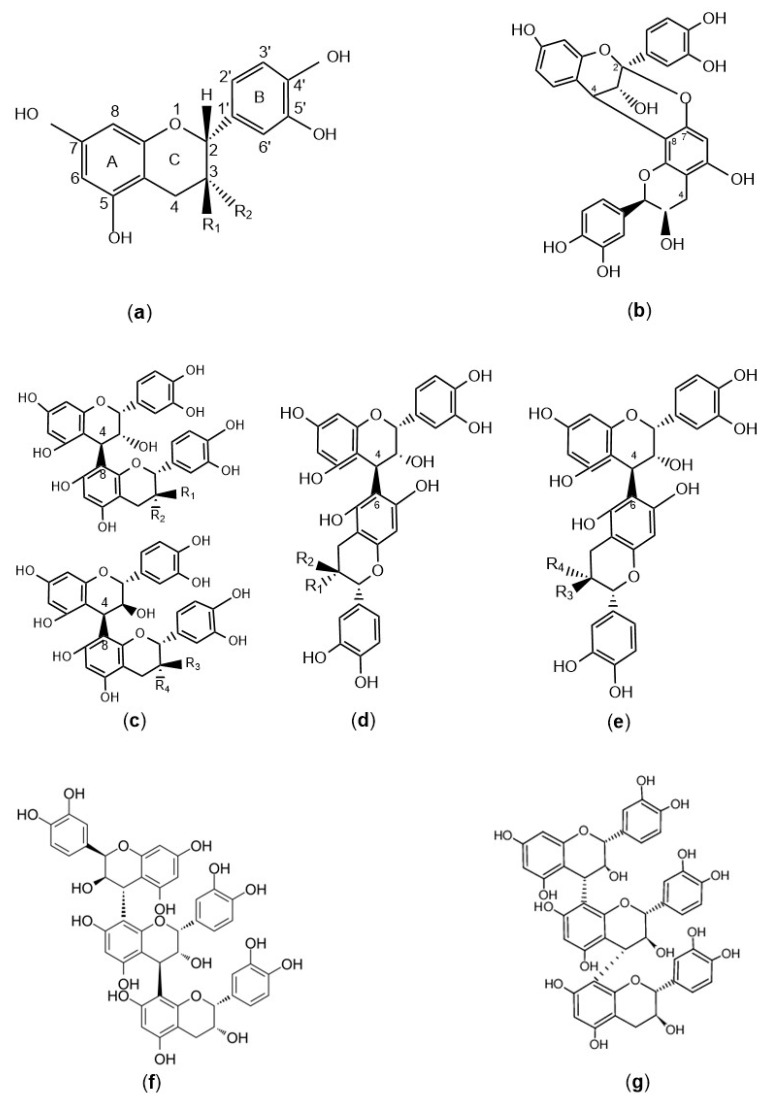
Chemical structure of PCs. (**a**) PC monomers; (**b**) PC A; (**c**) PCs B_1_-B_4_; (**d**) PCs B_5_-B_6_; (**e**) PCs B_7_-B_8_; (**f**) PC C_1_; (**g**) PC C_2_.

Proanthocyanidins, also known as condensed tannins (CTs), are oligomers or polymers composed of flavan-3-ol monomers. Under acidic conditions and heat, they yield anthocyanins. Depending on the degree of polymerization and monomer conformation, they are categorized as monomeric proanthocyanidins with a polymerization degree of 1, oligomeric proanthocyanidins (OPCs) with a polymerization degree ranging from 2 to 4, and polymeric proanthocyanidins (PPCs) with a polymerization degree exceeding 5. Based on the mode of monomer linkage, proanthocyanidins are further classified as type A proanthocyanidins, which feature C_4_-C_8_ or C_4_-C_6_ bonding and a single ether linkage of C_2_-O-C_7_ or C_2_-O-C_5_, or type B proanthocyanidins, which are characterized by C_4_-C_8_ or C_4_-C_6_ bonding [5]. Type C proanthocyanidins, on the other hand, possess a unique structure comprising one epicatechin monomer and two catechin monomers that are connected via C_4_-C_8_ and C_4_-C_6_ linkages to form a cyclic structure. The chemical structures of the different proanthocyanidins are depicted in Figure 1, and the types and molecular formulas are summarized in Table 1.

The biosynthesis of proanthocyanidins commences with phenylalanine, a fundamental amino acid prevalent in plants. This phenylalanine is enzymatically converted by phenylalanine lyase (PAL) into cinnamic acid, which subsequently undergoes further transformation into coumaroyl coenzyme A. These intermediates are then enzymatically processed by chalcone synthase (CHS) and chalcone isomerase (CHI), leading to the formation of anthocyanins. The anthocyanins are subsequently transformed into flavan-3-ol monomers that serve as the fundamental structural units of proanthocyanidins. Finally, a sequence of oxidative and polymerization reactions takes place, leading to the formation of proanthocyanidin polymers.

Within plants, proanthocyanidins serve multiple crucial functions. Firstly, they exhibit robust antioxidative properties, aiding plants in their resilience against environmental stressors such as UV radiation, drought conditions, and elevated temperatures. Additionally, proanthocyanidins bolster the disease resistance of plants, mitigating the deleterious effects of diseases by scavenging reactive oxygen radicals. Furthermore, they exert a beneficial influence on plant growth and development, facilitating cell division and elongation processes, thereby shaping the overall growth and morphology of the plant.

Proanthocyanidins have been extensively associated with diverse health benefits for humans. Their antioxidative properties are paramount in mitigating oxidative stress and inflammation, both of which are implicated in a range of chronic ailments, encompassing cardiovascular disorders, cancer, and neurodegenerative diseases. Furthermore, proanthocyanidins can improve vascular function, bolster skin health, and enhance cognitive capabilities. Consequently, these health-promoting effects render proanthocyanidins invaluable as components of the human diet, particularly in foods abundant in fruits, vegetables, and beverages, such as tea and red wine.

## 3. Extraction Techniques for Proanthocyanidins

In the extraction of proanthocyanidins, the selection of solvents holds a pivotal position in the isolation of specific compound types. These solvents exhibit distinct solubilities and affinities toward proanthocyanidins, thereby yielding variable extraction efficiencies. Consequently, when developing extraction protocols, it is imperative to meticulously consider the solubility and affinity of the target proanthocyanidins in various solvents. Commonly used solvents for proanthocyanidin extraction include methanol, ethanol, acetone, water, and mixtures thereof. The choice of solvent depends on the specific proanthocyanidins being targeted. For example, methanol and ethanol are effective solvents for extracting proanthocyanidins of low molecular weight, while acetone and water are more suitable for proanthocyanidins of higher molecular weight. In addition to the solubility and affinity of the solvents, other factors to consider when selecting a solvent include its toxicity, cost, and environmental impact. It is important to strike a balance between extraction efficiency and these other factors to optimize the extraction process.

Currently, the extraction of proanthocyanidins primarily involves two primary methods: solvent-based extraction and enzymatic hydrolysis. Solvent-based extraction efficiently extracts proanthocyanidins from plant materials, offering simplicity and cost-effectiveness. However, it is associated with issues such as environmental pollution and time-consuming procedures. To address these limitations, auxiliary extraction techniques such as ultrasonic-assisted, microwave-assisted, and high-voltage pulsed-electric-field-assisted methods have been continuously developed and implemented. These auxiliary techniques exhibit advantages like simplicity in operation, high extraction efficiency, and minimal environmental impact, leading to their widespread utilization in industrial settings. In contrast, enzymatic hydrolysis is more environmentally friendly and avoids solvent residue issues. Nevertheless, its operational process can be rather intricate, necessitating the meticulous control of reaction conditions.

In summary, different extraction techniques for proanthocyanidins possess distinct advantages and disadvantages, necessitating an approach tailored to specific scenarios. Additionally, given the significant variations in proanthocyanidin content among different plants and extraction sites, it is imperative to flexibly adjust extraction methods accordingly. As a crucial factor influencing extraction efficiency, the selection of solvents deserves thorough consideration and research.

### 3.1. Solvent Extraction

In the extraction of proanthocyanidins, the meticulous selection of solvents is paramount. Firstly, solvents must effectively solubilize the target compounds. Secondly, the polarity and safety profiles of solvents serve as crucial considerations. Typically, organic solvents, including methanol, ethanol, and acetone, are widely employed in the extraction of proanthocyanidins.

Liu et al. [6] successfully extracted proanthocyanidins from *Pennisetum purpureum* cv. Red using an acidic acetone solution, achieving a content of 5.06 mg/g with a minimal relative deviation of 0.198% from the theoretical extraction yield of 5.05 mg/g. Proanthocyanidins exhibit sensitivity to acidic environments, and their exposure to acidic conditions and heat can result in their conversion into anthocyanins. Consequently, the utilization of acidic acetone for the extraction of proanthocyanidins necessitates precise control of the pH and temperature to minimize this conversion process.

On the other hand, Hou et al. [7] adopted deep eutectic solvents for the extraction of proanthocyanidins from Camellia oleifera seed shells. The optimal conditions were determined to be an 80% choline chloride–citric acid aqueous solution, with a solid-to-liquid ratio of 1 g:200 mL at a temperature of 80 °C and an extraction time of 40 min. Under these optimized conditions, the extraction rate of proanthocyanidins reached 5.26%.

By carefully selecting appropriate solvents, proanthocyanidins can be rapidly and efficiently extracted from plants, thereby enhancing production efficiency and resource utilization. However, this process can result in the loss of thermally unstable components, and the utilization of organic solvents may pose potential risks, such as increased impurity levels in the extract and environmental pollution. Therefore, a balanced approach must be taken to maximize the extraction efficiency while minimizing these potential drawbacks.

#### 3.1.1. Ultrasonic-Assisted Extraction

Thilakarathna and Rupasinghe [8] demonstrated that the extraction of proanthocyanidins from grape seeds and grape seed powder utilizing an ethanol–aqueous solution at room temperature exhibits significant inefficiency and necessitates a considerable quantity of ethanol. However, the integration of ultrasound and heating into the extraction process has the potential to substantially shorten the extraction time and reduce the requirement for the ethanol–aqueous solution, thereby enhancing the yield of proanthocyanidins. Ultrasonic-assisted extraction employs ultrasonic waves to disrupt plant cell walls, accelerating the release of intracellular components and enhancing extraction efficiency. This method is renowned for its high efficiency, time savings, and simplicity. In addition, Wang [9] successfully applied ultrasonic waves in combination with solvents to extract proanthocyanidins from *Euterpe oleracea* (*açaí*) fruit. Through response surface methodology, the optimal extraction conditions were determined to be a 70% ethanol concentration, a solid-to-liquid ratio of 1 g:20 mL, an ultrasonic power of 180 W, a temperature of 75 °C, and an extraction time of 50 min. Under these conditions, the extraction yield reached 2.0278%. Huang et al. [10] utilized ultrasonic-assisted ethanol extraction to extract proanthocyanidins from the red hull of *Arachis hypogaea* (peanut). Through orthogonal experiments, the optimal conditions were found to be a solid-to-liquid ratio of 1 g:45 mL, a 60% ethanol concentration, an ultrasonic temperature of 35 °C, an ultrasonic time of 15 min, and a water bath temperature and time of 50 °C and 50 min, respectively. Under these conditions, the maximum extraction yield of proanthocyanidins from peanut hulls reached 9.07%.

The ultrasonic-assisted extraction method significantly reduces the extraction time compared with traditional single-solvent methods, greatly enhancing extraction efficiency and demonstrating promising application prospects. However, in practical applications, factors such as thermal and cavitation effects can easily lead to increased temperatures in the extraction equipment, potentially causing economic losses in the industrial extraction of thermally labile plant-derived substances.

#### 3.1.2. Microwave-Assisted Extraction

Compared with traditional extraction techniques, microwave-assisted extraction of proanthocyanidins offers the advantages of time savings, high extraction efficiency, and minimal destructiveness. Zhang et al. [11] employed microwave-assisted enzymatic hydrolysis to extract proanthocyanidins from grape (*Vitis vinifera* L.) seeds. Through response surface optimization, the optimal extraction conditions were determined to be a solid-to-liquid ratio of 1 g:15 mL, a cellulose addition of 0.56 g/L, an extraction temperature of 60 °C, and a microwave exposure time of 9 min. Under these conditions, the extraction yield of proanthocyanidins reached 5.72%. Liu et al. [12] utilized microwave-assisted solvent extraction to extract proanthocyanidins from camphor (*Cinnamomum camphora*) leaves. The optimal extraction conditions, obtained through response surface optimization, were an ethanol concentration of 77%, a solid-to-liquid ratio of 1 g:20 mL, a microwave exposure time of 18 min, and a microwave power of 530 W. Under these conditions, the extracted content of proanthocyanidins from camphor leaves was 81.56 ± 2.03 mg/g.

Currently, microwave-assisted extraction faces limitations in industrial production due to its restricted adaptability to laboratory research and the potential for high extraction forces to damage the structural integrity of target compounds. Therefore, the widespread application of microwave-assisted extraction remains limited.

#### 3.1.3. High-Intensity Pulsed Electric Field (PEF)-Assisted Extraction

High-intensity PEF-assisted extraction, based on the principle of electroporation, involves exposing cells to a high-voltage electric field. This process utilizes high-voltage direct current to transiently disrupt cell walls or membranes, resulting in intracellular potential disruption, altered cell permeability, and irreversible damage to cell walls and membranes, thereby facilitating the efflux of cellular components. Dong et al. [13] employed a PEF to extract proanthocyanidins from Amur grape (*Vitis amurensis*) seeds. Response surface optimization revealed that the optimal conditions for PEF-assisted extraction were a pulse count of 10, an electric field strength of 25 kV/cm, a solid-to-liquid ratio of 1 g:21 mL, and an ethanol concentration of 63% (*v*/*v*). Under these conditions, the yield of proanthocyanidins from Amur grape seeds reached 8.23%. A comparative analysis of the antioxidative activity of proanthocyanidins extracted using a PEF, ultrasonic-assisted extraction, microwave-assisted extraction, and ethanol extraction demonstrated that a PEF exhibited the highest extraction yield of proanthocyanidins. Notably, there were no significant differences in the total antioxidative capacity of proanthocyanidins obtained by the four methods. Furthermore, the DPPH^•^-radical scavenging activity and ferrous ion chelating ability of proanthocyanidins extracted using a PEF were comparable to those extracted by ultrasonic and microwave methods and superior to those extracted with ethanol, suggesting that PEF extraction is an effective method for extracting proanthocyanidins.

PEF extraction offers advantages such as non-thermal processing, short treatment duration, and high efficiency. Currently, this technology is primarily utilized in food processing, with relatively limited research on its application in the extraction of bioactive components from plants. Additionally, the complexity of circuit design and the high costs associated with PEF equipment have hindered its widespread industrial application.

### 3.2. Enzymatic Hydrolysis

Enzymatic hydrolysis employs enzymatic compounds such as cellulase, papain, and pectinase to disintegrate and disrupt plant cell walls, facilitating the solubilization of intracellular substances, thus significantly enhancing the extraction yield of proanthocyanidins. Wang et al. [14] utilized a combination of cellulase and pectinase to extract proanthocyanidins from *Dioscorea alata*. Through response surface optimization, the optimal extraction conditions were determined as follows: solid-to-liquid ratio of 1 g:15 mL, pH of 4.5, cellulase addition of 2.19%, pectinase addition of 2.32%, temperature of 45 °C, and duration of 73 min. Under these conditions, the yield of proanthocyanidins from *Dioscorea alata* was 93.06 mg/g, closely matching the predicted yield obtained from the response surface method. Huang et al. [15] similarly used a combination of pectinase and cellulase to extract proanthocyanidins from lotus seedpods. Orthogonal experiments revealed the optimal conditions as a pectinase-to-cellulase ratio of 1:1, a duration of 60 min, a temperature of 55 °C, and a pH of 6. Under these conditions, the extraction yield of proanthocyanidins from lotus seedpods was 4.36%.

While enzymatic hydrolysis boasts advantages such as high efficiency, safety, and specificity, it also faces drawbacks like high costs, complex processes, and limited reaction conditions, hindering its application in large-scale industrial production. In contrast, microbial fermentation extraction does not require strict control of the conditions, avoids the generation of significant industrial waste, and the resulting fermentation residue can be utilized as organic feed, offering broader development prospects. Ma et al. [16] employed a fermentation broth containing pectinase produced by *Paenibacillus polymyxa* to further extract proanthocyanidins from grape seeds. Through comprehensive orthogonal experiments, the optimal extraction conditions were identified as an enzyme activity of 313 U/mL from the *Paenibacillus polymyxa* fermentation broth, a solid-to-liquid ratio of 30 g:1 L, a duration of 70 min, an enzymatic hydrolysis temperature of 45 °C, and a pH of 9. Under these conditions, the extraction yield of proanthocyanidins from grape seeds reached 3.39%, significantly higher than the 0.64% yield achieved through water extraction, demonstrating the high efficiency and effectiveness of microbial fermentation extraction.

In conclusion, while various extraction techniques exhibit distinct advantages and disadvantages, it is imperative to thoroughly compare their overall yields to effectively inform practical industrial applications. Factors such as extraction efficiency and yield are meticulously analyzed to determine the best method for a particular industrial environment. Moreover, integrating the principles of different extraction methods, harnessing their synergistic effects and capitalizing on their complementary advantages are expected to enhance the extraction efficiency. This strategic approach aims to optimize processes for their widespread adoption in industrial production.

## 4. Methods for the Degradation of Proanthocyanidins

Monomers and oligomers of proanthocyanidins can be rapidly absorbed in vivo, whereas polymers with higher degrees of polymerization and larger molecular weights have difficulty penetrating cell membranes and entering cells [17]. Research has shown that OPCs exhibit superior antioxidative activity and inhibitory effects on xanthine oxidase compared with PPCs [18]. Compared with PPCs, OPCs possess higher biological activity and are more effective in the treatment of diabetes [19]. Therefore, to enhance the bioavailability and related biological activities of proanthocyanidins, they are often degraded into oligomers or monomers. The primary degradation methods for proanthocyanidins include acid-catalyzed hydrolysis, alkali degradation, hydrogenation degradation, oxidative degradation, and biodegradation.

### 4.1. Acid-Catalyzed Hydrolysis

The presence of nucleophilic reagents enables the cleavage of the C_4_-C_8_ bonds within the molecular structure of polymeric proanthocyanidins through thermal acidolysis, resulting in their depolymerization into OPCs. Luo et al. [20] employed 11% sulfurous acid to catalyze the degradation of PPCs from grape seeds and skins into OPCs. The degradation conditions for PPCs from grape seeds were as follows: 60 °C, 60 min, and a sample-to-sulfurous acid ratio of 1 mg:0.2 mL. The total degradation rate reached 43.94%. Since the content of proanthocyanidins in PPCs from grape seeds accounted for approximately 38.85%, it was demonstrated that PPCs in grape seeds could be completely converted into OPCs, with impurities accounting for approximately 5.09%. For PPCs from grape skins, the degradation conditions were 40 °C, 60 min, and a sample-to-sulfurous acid ratio of 1 mg:0.2 mL. The total degradation rate of PPCs from grape skins reached 25.14%. Given that the content of proanthocyanidins in PPCs from grape skins was approximately 22.46%, it was also shown that PPCs in grape skins could be fully converted into OPCs, with impurities accounting for approximately 2.68%.

Souza et al. [21] aimed to optimize the acid cleavage process of proanthocyanidins and other polyphenols extracted from plant matrices. Employing response surface methodology, they optimized the process using extracts from *Byrsonima crassifolia*, *Euterpe oleracea*, and *Inga edulis*. The study varied the hydrochloric acid concentration, time, and temperature to assess their effects on proanthocyanidins’ reduction, astringency, antioxidative capacity, and cyanidin content. Optimal conditions (3 N HCl, 88 °C, 165 min) resulted in a significant reduction in proanthocyanidins and astringency by 91% and 75%, respectively, across all extracts. The TEAC/TP ratio remained unchanged post-treatment, indicating an efficient reduction of undesirable proanthocyanidins characteristics. The increase in cyanidin content confirmed successful cleavage, suggesting broader industrial applications for acid cleavage in improving proanthocyanidin extract quality.

### 4.2. Alkali Degradation

Under certain temperatures, the C_4_-C_8_ bonds of PPCs are cleaved using alkaline reagents as catalysts to obtain OPCs. Ji et al. [22] compared the degradation effects of five different alkaline solutions—NaOH, Na_2_CO_3_, Na_2_SO_3_, NaHCO_3_, and NaHSO_3_—on polymeric proanthocyanidins from raspberry fruits. They found that Na_2_SO_3_ exhibited the best degradation performance. Under the conditions of a solid-to-liquid ratio of 1 g:10.25 mL, a Na_2_SO_3_ concentration of 2.13%, a reaction time of 42 min, and a temperature of 60 °C, the average degree of polymerization of proanthocyanidins decreased from 5.44 to 2.14 ± 0.11. Notably, the inhibitory effect of the degraded proanthocyanidins on hypoglycemic enzyme activity was significantly enhanced. Zhang et al. [23] explored the influence of different concentrations of sodium hydroxide on the depolymerization of grape seed proanthocyanidins. They discovered that under the conditions of 60 °C and 15 min, the use of 10 mol/L NaOH resulted in the best depolymerization effect, with the average degree of polymerization decreasing from 5.39 ± 0.12 to 1.30 ± 0.014.

### 4.3. Hydrogenation Degradation

The hydrogenation degradation method involves interfacial reactions in a heterogeneous gas–solid–liquid three-phase reaction system. The process proceeds as follows: H_2_ enters the liquid phase and is adsorbed onto the surface of a Pd/C catalyst, where it is dissociated into H under catalytic action. The H then participates in a bond-breaking reaction within the polymeric molecular structure, yielding catechin and epicatechin as reaction products. Jiang et al. [24] invented a method using ammonium formate as a hydrogen source, ethanol–water as a solvent, and Pd/C catalysis to reduce the average polymerization degree of proanthocyanidins from 68 to 23. This method offers advantages such as safe and efficient reactions, mild conditions, high yields, and low cost. Zhu et al. [25] employed PdCSO_3_H as a catalyst under conditions of 500 rpm stirring speed, a H_2_ pressure of 500 MPa, and a temperature of 220 °C for a reaction time of 2 h. This resulted in the average polymerization degree of proanthocyanidins being reduced from 7.60 to 3.14, with a depolymerization yield of 55.58%. Notably, the depolymerized OPCs exhibited superior antioxidative activity compared with PPCs.

### 4.4. Oxidative Degradation

Oxidative degradation serves as the primary means of proanthocyanidin degradation, a process that can be elicited by diverse factors encompassing exposure to light, heat, and oxygen. Once initiated, oxidative degradation gives rise to a range of products, encompassing anthocyanins, flavanols, and other compounds of low molecular weight.

Jorgensen et al. [26] conducted a study on the oxidative degradation of proanthocyanidins in grape skins and seeds under basic conditions. Their findings revealed that all the proanthocyanidin isolates underwent degradation, with grape skin proanthocyanidin exhibiting the most rapid degradation rate due to the presence of (−)-epigallocatechin extension subunits. Notably, the presence of flavan-3-alcohol monomer decelerated the degradation rate of grape seed proanthocyanidins. Furthermore, as the reaction progressed, the mean degree of polymerization (mDP) of proanthocyanidins decreased; however, the size distribution of proanthocyanidins remained relatively unchanged. This observation suggests that oxidative degradation of proanthocyanidins primarily occurs through the disruption of bonds between flavonoids rather than through the loss of individual flavonoid units.

### 4.5. Biodegradation

The biodegradation method can be classified into enzymatic biodegradation and microbial biodegradation. Enzymatic biodegradation utilizes enzymes that act on substrates similar to the structure and functional groups of proanthocyanidins, cleaving C-C single bonds. The crude enzyme solution obtained through fermentation then undergoes enzymatic hydrolysis reactions with PPCs, resulting in the production of OPCs. This method is characterized by its high efficiency, specificity, and mild reaction conditions. Microbial biodegradation, on the other hand, is based on the fact that proanthocyanidins are phenolic compounds with abundant polycyclic aromatic hydrocarbons in their structure. Microorganisms can open the benzene rings of PPCs, thereby forming OPCs.

Su et al. [27] were the first to utilize *N*-acetylneuraminic acid lyase for the degradation of proanthocyanidins. Under conditions of 50 °C, pH = 10, an enzyme addition of 0.35 mg/mL, and a reaction time of 8 h, the accumulation of proanthocyanidin monomers and dimers reached 35.67 mg/g and 14.49 mg/g, respectively, providing a new research direction for the depolymerization of PPCs. Wang [28] studied proanthocyanidins and found that under the action of *Aspergillus niger*, the polymerization degree of proanthocyanidins decreased from 6.80 to 4.53, demonstrating the ability of *Aspergillus niger* to depolymerize PPCs.

## 5. Biological Activities of Proanthocyanidins

**Figure 2:**
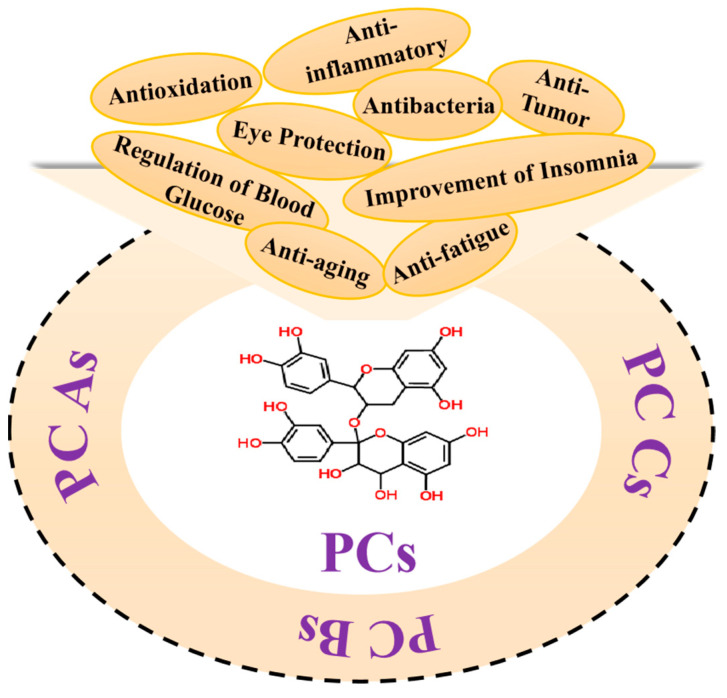
Proanthocyanidins’ biological activitities.

Due to its complex and diverse composition, proanthocyanidins exhibit various biological activities such as antioxidation, anti-inflammation, antitumor, and blood sugar regulation, as depicted in Figure 2.

### 5.1. Antioxidative Activity

Proanthocyanidins, as natural and efficient antioxidants, effectively scavenge free radicals and other oxidizing agents within the body, thereby preventing oxidative damage to cells and tissues. Additionally, they enhance cellular antioxidative defense capabilities and activate antioxidative enzyme systems [29]. Research on the antioxidative activity of proanthocyanidins primarily focuses on two aspects: exploring their antioxidative mechanisms, including the scavenging of free radicals and the activation of antioxidative enzyme systems; and evaluating their antioxidative potency through comparisons of the effects of different sources and types of proanthocyanidins in specific antioxidative tests.

In a study conducted by Xie et al. [30], the scavenging abilities of different polyphenol components from physiologically dropped litchi (*Litchi chinensis*) fruits against ABTS^+•^ and DPPH^•^ radicals, as well as their overall antioxidative capacity, were evaluated. The results revealed that pure polyphenol components, particularly type A proanthocyanidins, exhibited excellent antioxidative activities. Another study by Tan et al. [31] on proanthocyanidins from black goji berries demonstrated their ability to scavenge DPPH^•^ radicals and their antioxidative effects, establishing a positive correlation between antioxidative activity and concentration. Yu et al. [32] discovered that lotus seed pot proanthocyanidins (LSPCs), lotus seed pot oligomeric proanthocyanidins (LSOPCs), and lotus seed pot polymeric proanthocyanidins (LSPPCs) all exhibited a significant DPPH^•^ scavenging effect through in vitro free radical scavenging tests. Specifically, the IC_50_ values were determined to be 18.39 μg·mL^−1^, 6.56 μg·mL^−1^, and 20.77 μg·mL^−1^, respectively, which indicated that the LSOPCs have strong DPPH^•^ scavenging effects.

The antioxidative activity of proanthocyanidins is attributed to the presence of numerous phenolic hydroxyl groups, their specific molecular stereochemical structure, and the synergistic effect among the polymers. The degree of polymerization plays a pivotal role in determining the antioxidative activity of these compounds. Notably, proanthocyanidins with a low degree of polymerization exhibit stronger antioxidative activity. This is primarily due to the benzene ring containing a higher number of active phenolic hydroxyl groups, leading to superior antioxidative performance. However, as the degree of polymerization increases, the molecular interstitial gaps of proanthocyanidins become narrower, hindering the active role of hydroxyl groups. Consequently, the antioxidative activity of proanthocyanidins is limited by the narrower intermolecular space between the polymerized molecules.

Therefore, a thorough investigation of the antioxidative mechanisms of proanthocyanidins and a rigorous evaluation of their antioxidative activity not only deepen our understanding of their functional principles in the field of antioxidation but also provide scientific support for their applications in the food industry, drug development, and other fields. These achievements highlight the potential value of proanthocyanidins in promoting the health of organisms, opening new avenues for future research and application exploration.

### 5.2. Anti-Inflammatory Effects

Inflammation is an innate defensive mechanism of the body in response to various external stimuli, serving an essential protective function. However, when inflammation occurs improperly or excessively, it can trigger a series of health issues, highlighting the importance of the precise modulation of inflammatory responses in maintaining a healthy state.

Proanthocyanidins meticulously regulate inflammatory processes through multiple mechanisms, demonstrating their unique value and potential in the field of anti-inflammation. Specifically, they effectively suppress the production of key inflammatory mediators such as interleukin-6 (IL-6), interleukin-1β (IL-1β), and tumor necrosis factor-α (TNF-α). A study by Tao et al. [33] revealed that proanthocyanidin B2 suppresses inflammatory responses by regulating the activity of Sirt1/Sirt3, indicating its regulatory role at the molecular level. Furthermore, research conducted by Lu et al. [34] showed that proanthocyanidins can reduce the expression of NLRP3 inflammasome-related proteins in a dose-dependent manner and mitigate lipopolysaccharide (LPS)/adenosine triphosphate (ATP)-induced inflammatory responses and reactive oxygen species (ROS) levels by modulating key signaling pathways such as Toll-like receptor 4 (TLR4), myeloid differentiation factor 88 (MyD88), and nuclear factor kappa B (NF-κB).

Mansoor K. A. et al. [35] successfully isolated a novel proanthocyanidins trimer from *Cistus incanus*, specifically epigallocatechin-3-*O*-gallate-(4β→8)-epigallocatechin-3-*O*-gallate-(4β→8)-gallocatechin. In a COX inhibition test, this proanthocyanidin trimer exhibited an IC_50_ value of 4.5 μM against COX-2, which is significantly lower than that of a higher OPC fraction (IC_50_ 23.1 μM). This finding strongly suggests that the proanthocyanidins derived from *Cistus incanus* possess potent inhibitory activity against COX-2, thereby indicating their anti-inflammatory effects. OPCs exhibit better anti-inflammatory activity due to their smaller molecular weight, which enables it to pass through the cellular monomolecular layer and be absorbed and utilized more easily.

A comprehensive evaluation and analysis of research findings reveal the complexity of proanthocyanidins’ anti-inflammatory mechanisms. They not only directly suppress the release of key inflammatory mediators but also achieve a comprehensive regulation of inflammatory responses by acting on multiple aspects of inflammatory signaling pathways. The anti-inflammatory mechanisms of proanthocyanidins highlight their core roles in alleviating the tissue damage caused by inflammation, controlling the release of inflammatory mediators and modulating signaling pathways. These mechanisms provide a solid scientific foundation for their application in inflammation treatment and demonstrate their broad research and application prospects as effective anti-inflammatory components

### 5.3. Anti-Tumor Activity

Tumor development is a complex biological process encompassing oxidative stress, inflammatory responses, and the aberrant activation of molecular signaling pathways. Among these processes, proanthocyanidins, as naturally occurring antioxidants and anti-inflammatory agents, have demonstrated potential in suppressing tumorigenesis. Their effects extend beyond inhibiting tumor cell proliferation, invasion, and metastasis to promoting programmed cell death in tumor cells. These effects are achieved through the precise modulation of multiple molecular markers and signaling pathways, establishing a solid foundation for their future value in anti-tumor research and applications.

Su [36] discovered that proanthocyanidin B2 restores PTEN protein expression by reducing methylation in the PTEN gene promoter region, effectively suppressing tumor growth and reducing the tumor incidence. This not only highlights the role of proanthocyanidins in regulating the molecular mechanisms of tumor growth but also underscores the importance of the PTEN signaling pathway in tumor suppression. Furthermore, the elucidation of the regulatory mechanisms of proanthocyanidins provides a new perspective for understanding their anti-tumor activity and the scientific rationale for the development of targeted anti-tumor treatment strategies.

Jiang [37] conducted an in-depth investigation into the effects of procyanidins with varying polymerization degrees on hepatocellular carcinoma cells. The findings revealed that polymeric proanthocyanidins with an mDP greater than 5 possess the remarkable ability to significantly inhibit the proliferation and induce the apoptosis of these cancerous cells. Furthermore, this study demonstrated that polymeric proanthocyanidins regulate the expression of apoptosis-related proteins, including Bcl-2/Bax, Fas/FADD, and TNF-α, as well as modulate the activity of Caspase family proteins, thus revealing the mechanism of their inhibition of tumor growth. This important finding provides strong evidence for the potential of proanthocyanidins in clinical applications.

Research on triple-negative breast cancer has shown that proanthocyanidins specifically inhibit the proliferation of these tumor cells without significant effects on normal cells. This effect may be achieved through the inhibition of the Akt/mTOR/STAT3 signaling pathway [38], demonstrating the great potential of proanthocyanidins in the treatment of specific tumor types. This finding not only validates the possibility of proanthocyanidins as effective anti-tumor molecules but also provides new directions for treatment strategies targeting specific cancers.

The analysis and evaluation of the anti-tumor activity and mechanism of proanthocyanidins clearly demonstrate their role in regulating specific molecular markers and signaling pathways, influencing tumor cell growth and death processes. These research findings provide a solid scientific basis for their further application and development in the field of anti-tumor therapy. Future exploration and a deepening of our understanding of the anti-tumor potential of proanthocyanidins will contribute to the development of more effective treatment methods, bringing hope and potential therapeutic breakthroughs to patients with cancer.

### 5.4. Regulation of Blood Glucose

Diabetes, a widespread metabolic disease, is characterized by persistent hyperglycemia and either insufficient insulin production or dysfunction. This prolonged hyperglycemic state can lead to serious complications, such as atherosclerosis, myocardial infarction, and stroke, posing a significant threat to patients’ quality of life. In recent years, the search for natural modulators of blood glucose has gained momentum, with proanthocyanidins emerging as a promising candidate.

Proanthocyanidins exhibit their hypoglycemic effects through multiple mechanisms. Firstly, they improve glucose and lipid metabolism, thus enhancing insulin sensitivity and boosting antioxidative capacity. For instance, studies have shown that the administration of proanthocyanidins extracted from certain fruits, such as raspberries, can lead to significant improvements in insulin sensitivity and glucose control while reducing oxidative stress [39].

Furthermore, proanthocyanidins directly regulate blood glucose levels by inhibiting intestinal glucose production and transport, as well as by modulating the activity of enzymes involved in carbohydrate metabolism [40]. This direct action on glucose homeostasis provides a solid foundation for the use of proanthocyanidins in diabetes treatment.

Beyond their hypoglycemic effects, proanthocyanidins also show promise in preventing and mitigating diabetic complications. Research has demonstrated that these compounds can protect vital organs, such as the kidneys, from the damaging effects of diabetes [41]. This added benefit further enhances the therapeutic potential of proanthocyanidins in managing this chronic disease.

In conclusion, proanthocyanidins, as natural blood glucose modulators, offer a promising alternative for the treatment of diabetes. Their diverse mechanisms of action and ability to prevent diabetic complications make them a valuable addition to the armamentarium of therapeutic options for patients with diabetes. With further research, these compounds may pave the way for more effective and natural approaches to managing this widespread metabolic disease.

### 5.5. Antibacterial Activity

Proanthocyanidins have demonstrated broad antibacterial activity against a range of bacteria, including *Staphylococcus aureus* (*S. aureus*), *Escherichia coli* (*E. coli*), and *Salmonella* spp. [42]. The mechanism underlying this antibacterial activity is presumably linked to the interaction of proanthocyanidins with microbial cell membranes and walls. By disrupting bacterial cell wall structures and enhancing membrane permeability, proanthocyanidins effectively inhibit bacterial growth and reproduction. Additionally, they further suppress bacterial growth by interfering with their metabolic pathways [43].

When considering the antibacterial effects of proanthocyanidins, it is noteworthy that different bacteria exhibit varying sensitivities to this compound. For instance, Zhang [11] revealed that grape seed proanthocyanidins have a more profound inhibitory effect on *S. aureus* compared with *E. coli*. This observation underscores the importance of considering bacterial sensitivity when utilizing proanthocyanidins as antibacterial agents. Furthermore, the study demonstrated that proanthocyanidins damage the cell wall of *S. aureus*, increasing membrane permeability, and this antibacterial effect intensifies with increasing concentrations, providing direct evidence for the compound’s mechanism of action.

Complementing this research, Zhang’s study [44] further corroborated the inhibitory effects of proanthocyanidins on Gram-positive bacteria, emphasizing the crucial role of their concentration in determining antibacterial efficacy. This finding is pivotal in exploring the effective concentration range of proanthocyanidins for practical applications.

As a naturally abundant and safe compound, the remarkable antibacterial activity of proanthocyanidins signifies their potential application value in the fields of food safety and medical hygiene. Given their ability to effectively combat a wide range of bacteria, proanthocyanidins represent a promising candidate for the development of novel antibacterial agents.

### 5.6. Eye Protection

The retina, as a crucial neural tissue for visual transduction, plays a pivotal role in detecting and transmitting light signals to the brain [45]. Owing to its intricate structure, the retina is particularly susceptible to various harmful factors, such as neuroglial hyperplasia, ischemia, and oxidative stress. These factors can damage retinal cells, triggering multiple ocular diseases, including age-related macular degeneration and diabetic retinopathy, which significantly impact vision [46,47,48,49,50].

Research has demonstrated the significant potential of proanthocyanidins in preventing and treating ocular diseases. Wang et al. [51] discovered that lotus seedpod proanthocyanidins (LSPCs) effectively prevent light-induced retinal damage. Specifically, LSPCs significantly improved the thickness of the outer nuclear layer of the retina, reduced the elevation of lipid peroxide (MDA), nitric oxide (NO), and nitric oxide synthase (NOS) levels induced by light exposure, and attenuated the decrease in oxidative stress-related enzyme activities, such as glutathione peroxidase and superoxide dismutase (SOD). Furthermore, by modulating the expression of apoptosis-related proteins, LSPCs reduced light-induced retinal cell apoptosis and inhibited the overexpression of glial fibrillary acidic protein, resulting in comprehensive antioxidative, anti-apoptotic, and neuroprotective effects.

In studies on diabetic retinopathy, Tang et al. [52] observed the effects of proanthocyanidins on human retinal capillary endothelial cells (HRCECs) under hyperglycemic conditions. They found that proanthocyanidins significantly inhibited the proliferation of HRCECs and reduced the expression of the vascular endothelial growth factor. This effect was positively correlated with concentration and enhanced over time, suggesting that proanthocyanidins reduce the risk of diabetic retinopathy by suppressing the excessive proliferation and angiogenesis of HRCECs in hyperglycemic environments.

These research findings not only validate the application potential of proanthocyanidins in eye protection but also reveal the complexity of their underlying mechanisms. By antagonizing oxidative stress, regulating apoptotic pathways, and suppressing pathological angiogenesis, proanthocyanidins offer a multifaceted strategy for preventing and treating related retinal diseases.

### 5.7. Anti-Aging Properties

Aging, a complex process influenced by genetics, environment, and lifestyle, is characterized by the gradual decline of physiological and psychological functions. It is associated with cellular and molecular damage, metabolic disorders, and immune decline, leading to various health issues and compromising the quality of life.

Proanthocyanidins have been shown to possess anti-aging properties. Xu et al. [53] investigated the anti-aging activity of proanthocyanidin C1 and its role in extending the lifespan of mice. Their results revealed that at low concentrations, proanthocyanidin C1 suppressed the formation of senescence-associated secretory phenotypes, while at higher concentrations, it selectively eliminated senescent cells in a dose-dependent manner. Further studies demonstrated that proanthocyanidin C1 induced apoptosis in senescent cells by upregulating the pro-apoptotic factors NOXA and PUMA, enhancing reactive oxygen species production and causing mitochondrial dysfunction. Additionally, research has indicated that proanthocyanidin B2 protects against D-galactose-induced aging [54]. Proanthocyanidin B_2_ enhances anti-aging capabilities primarily through the regulation of various metabolic pathways, including the tricarboxylic acid cycle, fatty acid transport, unsaturated fatty acid synthesis, saturated fatty acid metabolism, and bile acid metabolism. Furthermore, it maintains the balanced proportion of intestinal bacterial structures in aging mice by modulating their gut microbiota, contributing to the enhancement of anti-aging activity.

Collectively, these findings highlight the anti-aging potential of proanthocyanidins, which offer a multifaceted approach to addressing the complexities of the aging process. By targeting various physiological mechanisms, these compounds may represent a promising avenue for the development of novel anti-aging strategies.

### 5.8. Anti-Fatigue Properties

Fatigue, a common outcome of intense physical activity, arises when the body’s aerobic metabolism cannot meet its energy demands, leading to anaerobic glycolysis. This process generates lactic acid, altering muscle pH and triggering biochemical reactions that promote free radical production and lipid peroxidation. Fatigue not only impairs physical performance but also affects mental well-being, potentially leading to psychological issues.

To investigate the anti-fatigue activity of proanthocyanidins, Zang et al. [55] compared various physiological and biochemical markers in exercised mice treated with proanthocyanidins to those in a control group. The treated mice exhibited improved serum creatine kinase activity, serum urea nitrogen levels, and malondialdehyde levels in liver tissue. Additionally, proanthocyanidins enhanced the activity of total superoxide dismutase and glutathione peroxidase in liver tissue while suppressing the overexpression of *Bax* and promoting *Bcl-2* gene expression. Notably, the anti-fatigue effects of proanthocyanidins were dose-dependent.

The above findings demonstrated that proanthocyanidins may be potential drugs for the treatment of fatigue-related problems. However, further studies are needed to fully elucidate their mechanism of action and potential clinical applications.

### 5.9. Improvement of Insomnia

Insomnia, a common non-respiratory sleep disorder, is characterized by difficulties in falling asleep and maintaining sleep, or by the experience of premature awakenings. Its detrimental effects are widespread, ranging from compromising the normal function of the immune system and reducing bodily resistance, thereby increasing the risk of illnesses such as colds and influenza, to adversely affecting the cardiovascular system and heightening the likelihood of developing heart disease and stroke. Furthermore, insomnia frequently coexists with mental health issues like anxiety and depression, potentially leading to long-term impairments in memory and learning abilities, and, in severe cases, even disrupting daily life and work.

Amidst the ongoing research to alleviate insomnia, proanthocyanidins have garnered significant attention due to their notable effects. Studies have demonstrated that proanthocyanidins effectively ameliorate insomnia and enhance sleep quality. Notably, Xiao et al. [56] conducted a thorough investigation into the inhibitory effects and underlying mechanisms of LSPC B_2_ on insomnia through the NO/ADMA/DDAH pathway. By comparing normal rats with insomnia rats treated with LSPC B_2_ for seven days through gastric lavage, they observed several positive changes in the treated rats: reduced walking time and forelimb lifting frequency; decreased levels of norepinephrine, glutamic acid, ADMA, sleep latency, and 8-iso-prostaglandin in the brain; and, concurrently, prolonged sleep duration, increased concentrations of 5-hydroxytryptamine, nitric oxide, and gamma-aminobutyric acid in the brain as well as the upregulated expression of DDAH1, DDAH2, and neuronal nitric oxide synthase. These findings suggest that LSPC B_2_ may effectively alleviate insomnia symptoms by suppressing oxidative stress and modulating the NOADMA/DDAH pathway.

In conclusion, the impact of insomnia on human health cannot be overstated, and proanthocyanidins, particularly LSPC B_2_, have demonstrated promising potential in the treatment of insomnia. Further exploration of its mechanisms is expected to lead to the development of more effective and safer therapeutic strategies for insomnia patients. Nevertheless, additional clinical studies are necessary to comprehensively assess their safety and long-term outcomes.

## 6. Conclusions and Outlook

This article explores various aspects of a natural product, proanthocyanidins, encompassing its extraction and purification techniques, degradation methods, biological activities, and underlying mechanisms. The findings reveal the remarkable research value and market potential of proanthocyanidins due to their unique physiological activities and diverse application domains. Nevertheless, current research still faces several challenges and limitations.

Firstly, in terms of extraction processes, despite numerous reported methods for extracting proanthocyanidins, most face issues such as low efficiency, insufficient purity, and environmental unfriendliness. To overcome these limitations, future research should prioritize optimizing existing extraction methods. This includes refining extraction solvents, optimizing extraction conditions (e.g., temperature, time, pH), and integrating modern separation techniques like supercritical fluid extraction and membrane separation. Such efforts would enhance the extraction efficiency and purity of proanthocyanidins while minimizing the environmental impact.

Secondly, the degradation methods and mechanisms of proanthocyanidins remain understudied. To gain a deeper understanding of their metabolic pathways and potential applications, future research should focus on elucidating their degradation kinetics, exploring the key factors influencing this degradation, and clarifying their regulatory mechanisms. This will aid to better predict and control the stability and biological activity of proanthocyanidins in vivo, providing a theoretical basis for their application in medicine, cosmetics, and other fields.

Regarding biological activities and mechanisms, while proanthocyanidins have been shown to possess various biological activities, including antioxidative, anti-inflammation, and antitumor effects, the specific mechanisms underlying these actions remain incompletely understood. Future research should leverage advanced molecular biology and cell biology techniques to delve deeper into the interactions between proanthocyanidins and the specific targets or signaling pathways within biological systems, as well as their functional mechanisms under various physiological and pathological conditions. This will facilitate a more comprehensive understanding of the biological functions of proanthocyanidins, providing scientific evidence for their application in disease prevention and treatment.

Lastly, in terms of applications, proanthocyanidins, as natural products with diverse biological activities, hold immense potential in functional foods, cosmetics, and pharmaceuticals. Future research should actively explore the specific applications and effects of proanthocyanidins in these areas. Through market expansion and brand building, consumer awareness and acceptance of proanthocyanidins can be enhanced, driving its industrialization process. Simultaneously, we must also pay attention to the safety and effectiveness evaluation of proanthocyanidins to ensure its safe and effective application in practice.

In summary, although progress has been made in the research on proanthocyanidins, numerous gaps and challenges remain. Future studies should focus on optimizing their extraction processes, elucidating their degradation mechanisms, revealing their biological activities and mechanisms, and exploring new application areas. By fully leveraging the potential of proanthocyanidins, we can contribute significantly to human health and well-being.

## Figures and Tables

**Table 1:** The type and molecular formulas of PCs.

PC Type		Functional Group	Molecular Formula
PC monomer	Catechin	R_1_ = OH, R_2_ = H	C_15_H_14_O_6_
	Epicatechin	R_1_ = H, R_2_ = OH	C_15_H_14_O_6_
	Catechin gallate	R_1_ = Gallate, R_2_ = H	C_22_H_18_O_10_
	Epicatechin gallate	R_1_ = H, R_2_ = Gallate	C_22_H_18_O_10_
PC A		—	C_30_H_24_O_12_
PC B_1_		R_1_ = OH, R_2_ = H	C_30_H_26_O_12_
PC B_2_		R_1_ = H, R_2_ = OH	
PC B_3_		R_3_ = OH, R_4_ = H	
PC B_4_		R_3_ = H, R_4_ = OH	
PC B_5_		R_1_ = OH, R_2_ = H	
PC B_6_		R_1_ = H, R_2_ = OH	C_30_H_26_O_12_
PC B_7_		R_3_ = OH, R_4_ = H	
PC B_8_		R_3_ = H, R_4_ = OH	
PC C_1_		—	C_45_H_38_O_18_
PC C_2_		—	

## Data Availability

Data supporting the reported results are available in the referenced literature.
